# Metabolomic analysis of uterine serous carcinoma with acquired resistance to paclitaxel

**DOI:** 10.18632/oncotarget.25868

**Published:** 2018-08-10

**Authors:** Manabu Seino, Tsuyoshi Ohta, Akiko Sugiyama, Hirotsugu Sakaki, Takeshi Sudo, Seiji Tsutsumi, Shogo Shigeta, Hideki Tokunaga, Masafumi Toyoshima, Nobuo Yaegashi, Satoru Nagase

**Affiliations:** ^1^ Department of Obstetrics and Gynecology, Yamagata University School of Medicine, Iidanishi, Yamagata 990-9585, Japan; ^2^ Department of Obstetrics and Gynecology, Tohoku University Graduate School of Medicine, Iidanishi, Yamagata 990-9585, Japan

**Keywords:** endometrial cancer, uterine serous carcinoma, metabolomic analysis, paclitaxel

## Abstract

**Introduction:**

Uterine serous carcinoma (USC) is more aggressive than other subtypes of endometrial carcinoma and is associated with a poor prognosis. We analyzed the metabolomic profile of USC with acquired resistance to paclitaxel.

**Results:**

Glutathione (GSH) concentration in PTX-1 cells was higher than in USPC-1 cells. In addition, GSH concentration in the USPC-1 cells increased after treatment with paclitaxel but was unchanged in PTX-1 cells. Glucose-6-phosphate (G6P) and ribose-5-phosphate (R5P) concentrations in PTX-1 cells were higher than those in USPC-1 cells. G6P concentration in the USPC-1 cells was unchanged after treatment with paclitaxel, while it decreased in PTX-1 cells.

**Conclusion:**

Our results indicate that increased GSH and glucose metabolism may be related to acquiring resistance to paclitaxel in USC and thus may be targets for anti-USC therapy.

**Materials and Methods:**

We compared metabolic profiles and reactions to paclitaxel in both a wild type USC cell line (USPC-1) and PTX-1, a cell line derived from USPC-1 which acquired paclitaxel resistance, using a capillary electrophoresis CE-MS/MS system.

## INTRODUCTION

Endometrial carcinoma is a common gynecologic malignancy in women. Most endometrial cancers are classified as early stage and low grade (i.e., endometrioid carcinoma grade 1, 2), with a 5-year survival of greater than 85% [1]. However, the rare histologic type uterine serous carcinoma (USC) is more aggressive than other subtypes and is associated with a poor prognosis. The proportion of USC is less than 10% of all subtypes of endometrial carcinoma, but the 5-year survival of USC is poor with only 18–27%, compared to that of low-grade types [1–3]. Type I endometrial carcinomas comprise endometrioid carcinomas (grade 1, 2), are usually seen in younger patients, and are associated with obesity, hyperlipidemia, and hyperestrogenism. In contrast, Type II endometrial carcinomas include serous carcinoma and clear cell carcinoma, are seen in older patients, and are not associated with hormonal factors.

As many as 37–70% of USC cases showed extrauterine disease at time of diagnosis [4, 5], sometimes with scant or no myometrial invasion [6, 7]. Most cases of USC relapse after initial treatment, like epithelial ovarian serous carcinomas. The rate of recurrence is estimated to be 31–80% [4, 8, 9]. Some studies suggested a beneficial role for adjuvant chemotherapy in USC even when diagnosed at an early stage [10–12]. However, many cases with USC received chemotherapy (paclitaxel/carboplatin) and as in cases of ovarian cancer, some acquired resistance to chemotherapy and had poor prognosis. Tubulin-β-III overexpression is a marker for poor prognosis after platinum/taxane chemotherapy [13], but other mechanisms leading to chemoresistance in USC are unclear.

Metabolomic analysis is a new technique for evaluation of biological specimens to reveal various metabolic pathways. Metabolites are the end products of metabolic pathways and may be involved in various tumor features. Targeting metabolic enzymes from key metabolic pathways, like glucose metabolism, glutaminolysis and fatty acid synthesis, has been shown to enhance the cytotoxicity of various chemotherapeutic agents. It may be possible to improve the outcomes of chemoresistant tumors significantly by metabolite analysis [14, 15].

A metabolomic approach revealed parts of the mechanism for platinum resistance in ovarian cancer [16], but there has been no metabolomics study concerning USC specifically.

In many advanced cases of USC, chemotherapeutic agents including paclitaxel were administered, and some cases acquired resistance to repeated chemotherapy. We thus performed metabolomic analysis of USC cells with acquired resistance to paclitaxel. Our data suggest that both glutathione and glucose metabolism may play a role in the resistance of USC to paclitaxel.

## RESULTS

### Metabolomic analysis of USC cells

**Figure 1:**
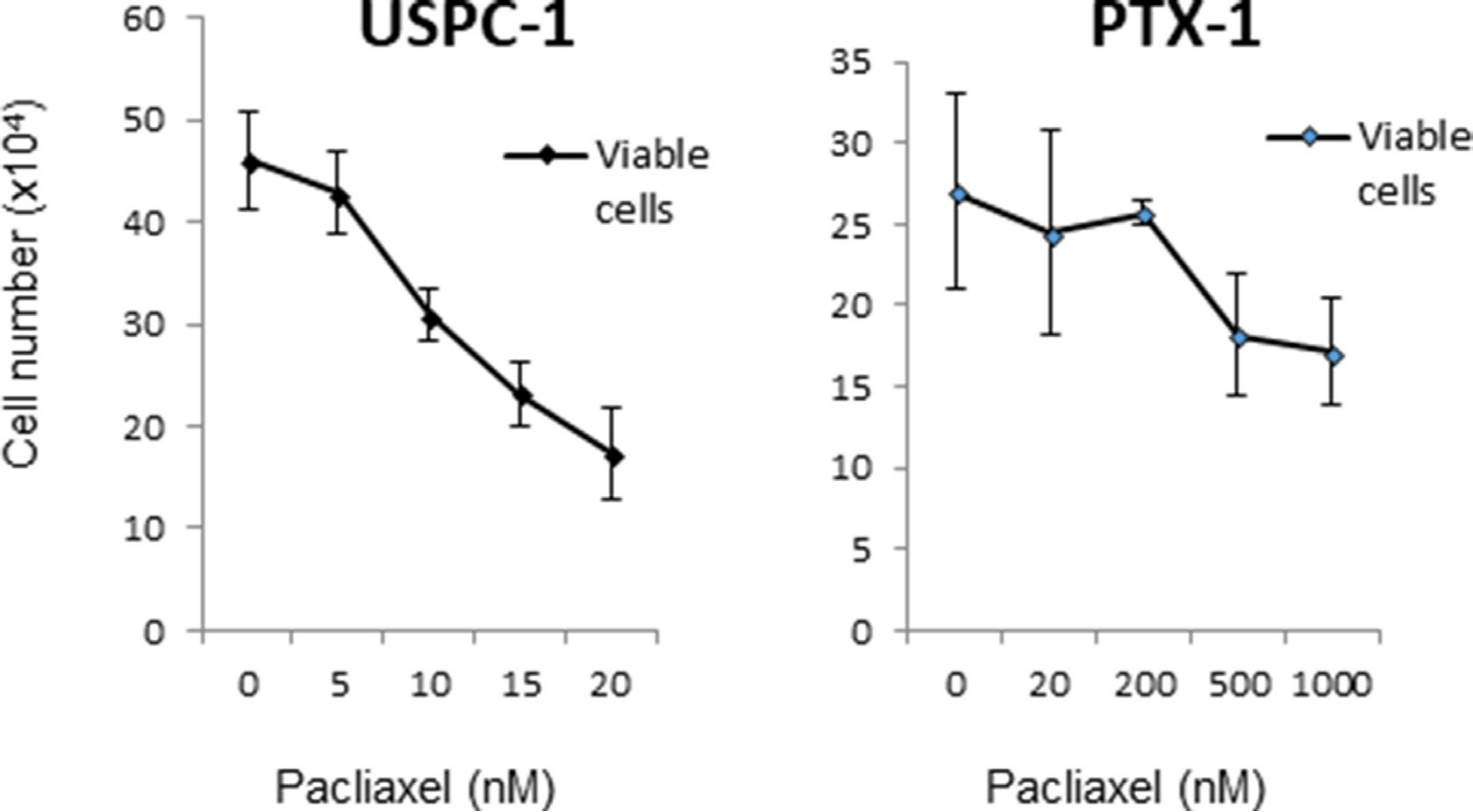
Growth inhibition of uterine serous carcinoma cells after treatment with paclitaxel USPC-1 and PTX-1 cells were treated with the indicated concentrations of paclitaxel for 24 h. Then the number of viable cells was determined for each cell line. The IC_50_ concentration was calculated using the formula described in Materials and Methods. Values in the graphs represent the means ± SD of three independent experiments.

We first confirmed resistance to paclitaxel in USPC-1 and PTX-1 cells and found that PTX-1 cells were significantly resistant to paclitaxel compared to USPC-1 cells (Figure 1). The proportion of PTX-1 cells in the G2/M phase was greater than that of USPC-1 cells (Supplementary Figure 1).

Next, we performed metabolomic analysis of USPC-1 and PTX-1 cells. The metabolic pathways assessed were glycolysis and pentose phosphate pathway, tricarboxylic acid cycle, urea cycle, the polyamine creatine metabolic pathway, purine metabolism, methionine cycle, glutathione (GSH) cycle, branched chain amino acid metabolism, lysine, tryptophan and nicotinamide metabolism, choline and fat metabolism.

**Figure 2:**
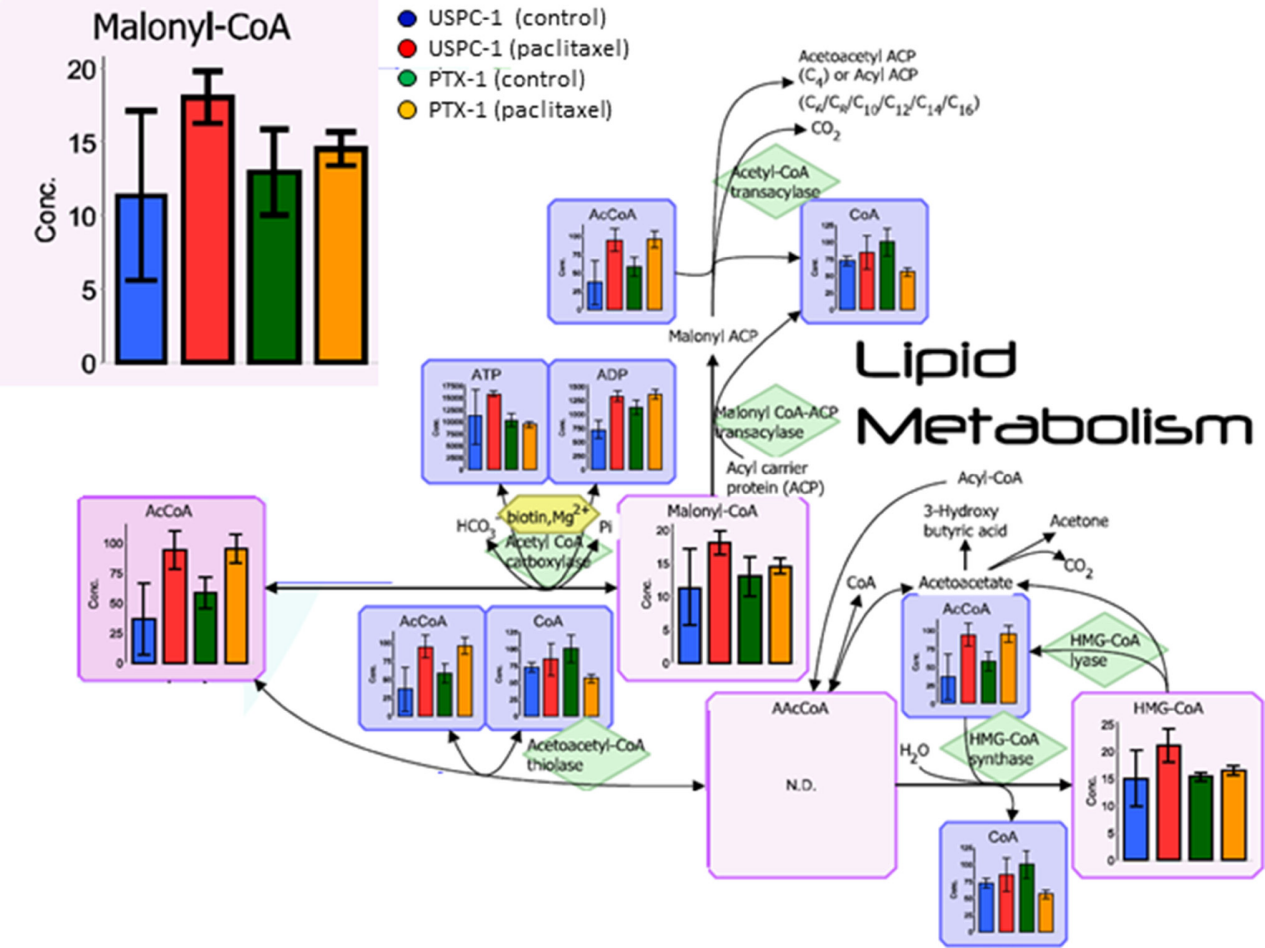
Lipid metabolism analysis after treatment with paclitaxel in uterine serous carcinoma cells Each cell line was treated with 15 nM paclitaxel or control vehicle for 24 h. Blue bars represent USPC-1 cells (control), red bars represent USPC-1 cells treated with paclitaxel, green bars represent PTX-1 cells (control) and yellow bars represent PTX-1 cells treated with paclitaxel. Values in the graphs represent the means ± SD of three independent experiments. ^*^P < 0.05. N.D.: not detected.

**Figure 3:**
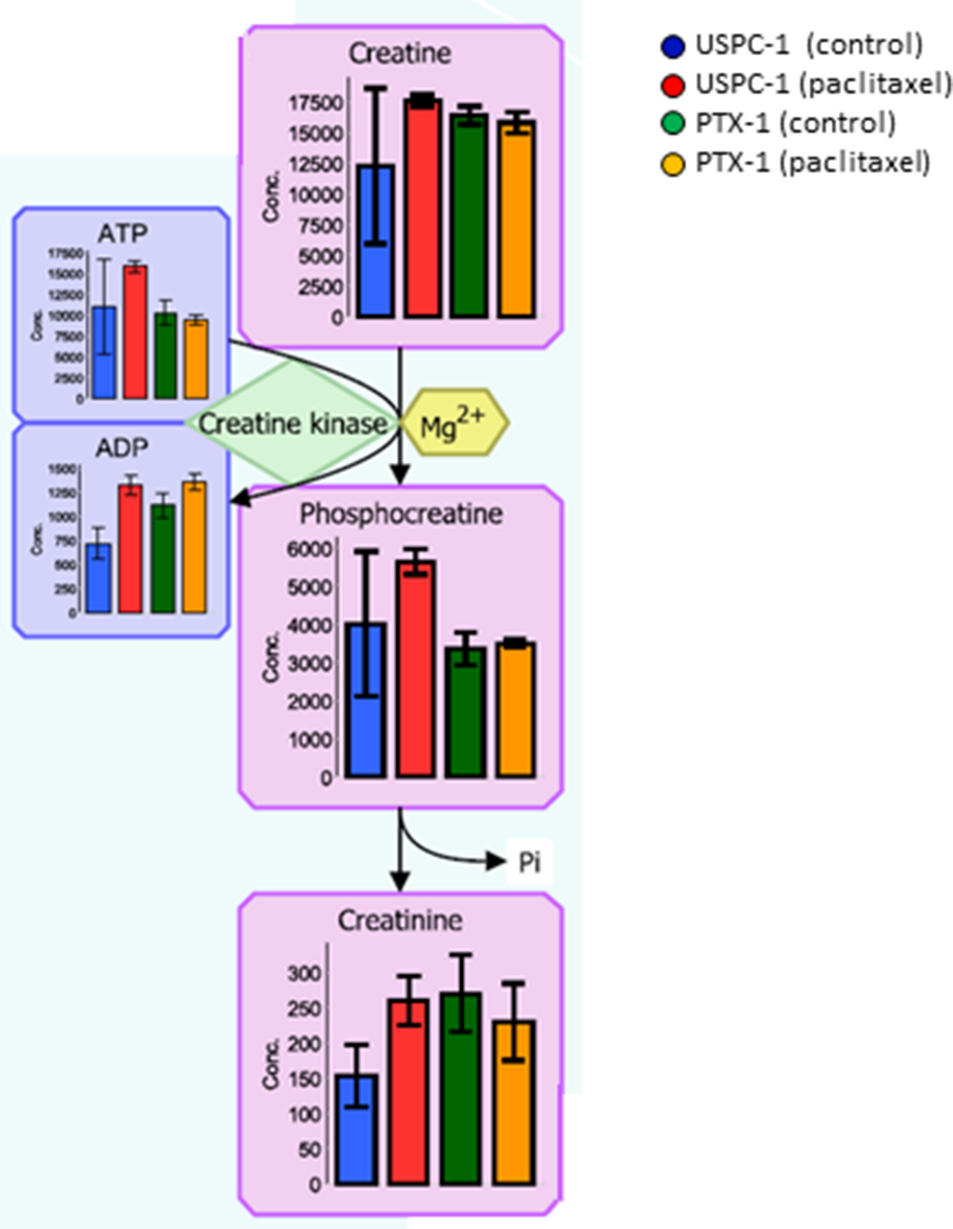
Creatine metabolism analysis after treatment with paclitaxel in uterine serous carcinoma cells Each cell line was treated with 15 nM paclitaxel or control vehicle for 24 h. Blue bars represent USPC-1 cells (control), red bars represent USPC-1 cells treated with paclitaxel, green bars represent PTX-1 cells (control) and yellow bars represent PTX-1 cells treated with paclitaxel. Values in the graphs represent the means ± SD of three independent experiments. ^*^P < 0.05.

**Figure 4:**
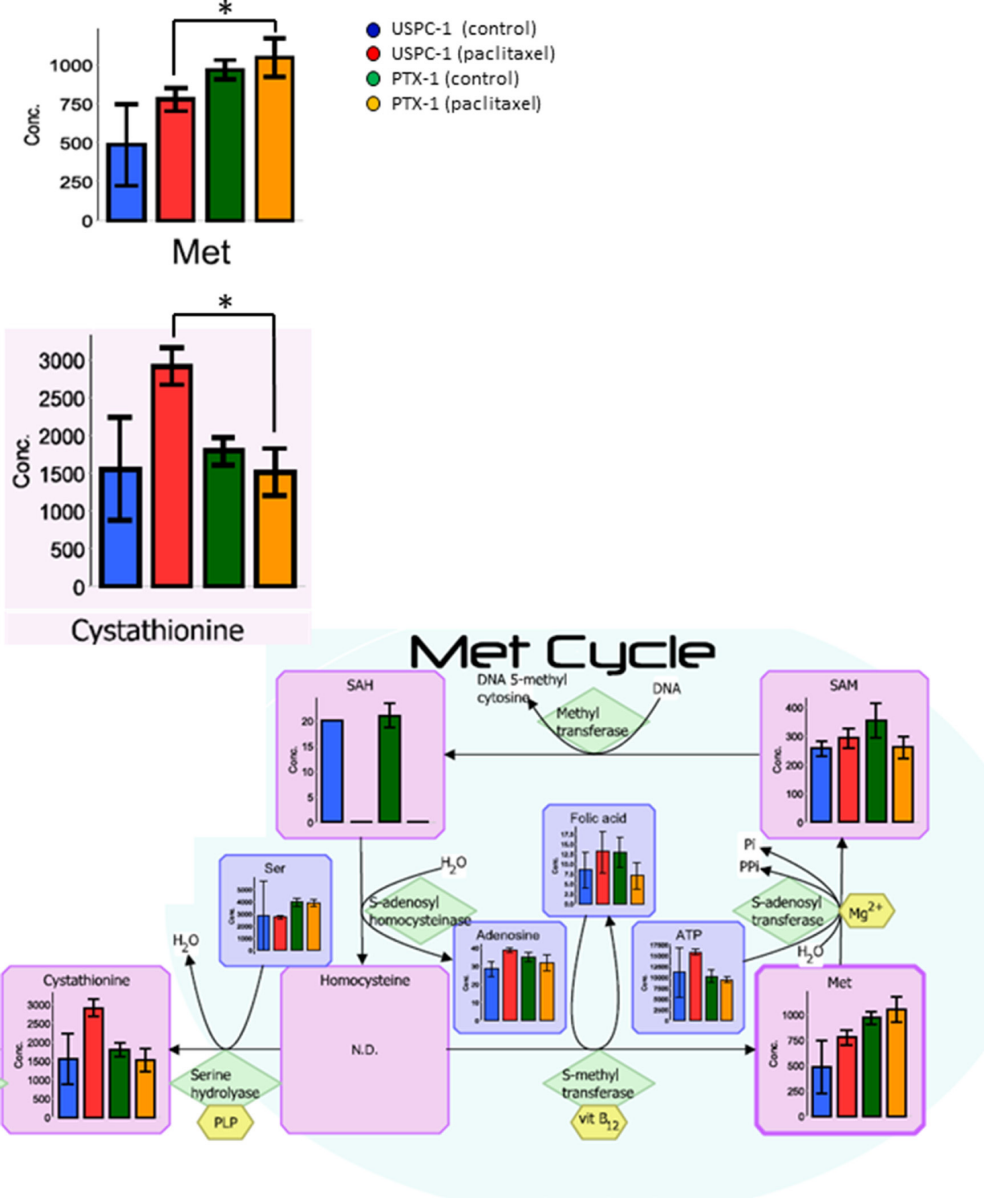
Methionine metabolism analysis after treatment with paclitaxel in uterine serous carcinoma cells Each cell line was treated with 15 nM paclitaxel or control vehicle for 24 h. Blue bars represent USPC-1 cells (control), red bars represent USPC-1 cells treated with paclitaxel, green bars represent PTX-1 cells (control) and yellow bars represent PTX-1 cells treated with paclitaxel. Values in the graphs represent the means ± SD of three independent experiments. ^*^P < 0.05. N.D.: not detected.

In our analysis, we focused lipid metabolism, polyamine and creatine metabolism, methionine cycle, glutathione (GSH) metabolism and glucose metabolism. Malonyl-coenzyme-A (CoA) concentration in the lipid metabolism of USPC-1 cells, but not PTX-1 cells, was elevated after treatment with paclitaxel (Figure 2). Similarly, creatine, phosphocreatine and creatinine concentrations were also elevated in USPC-1 cells, but not PTX-1 cells, after treatment with paclitaxel (Figure 3). Methionine concentration in USPC-1 cells is elevated after paclitaxel treatment, but not in PTX-1 cells (Figure 4). Methionine concentration in the PTX-1 cells after paclitaxel treatment was significantly higher than that of USPC-1 cells. Additionally, cystathionine levels in PTX-1 cells were significantly lower than those in USPC-1 cells after paclitaxel treatment.

**Figure 5:**
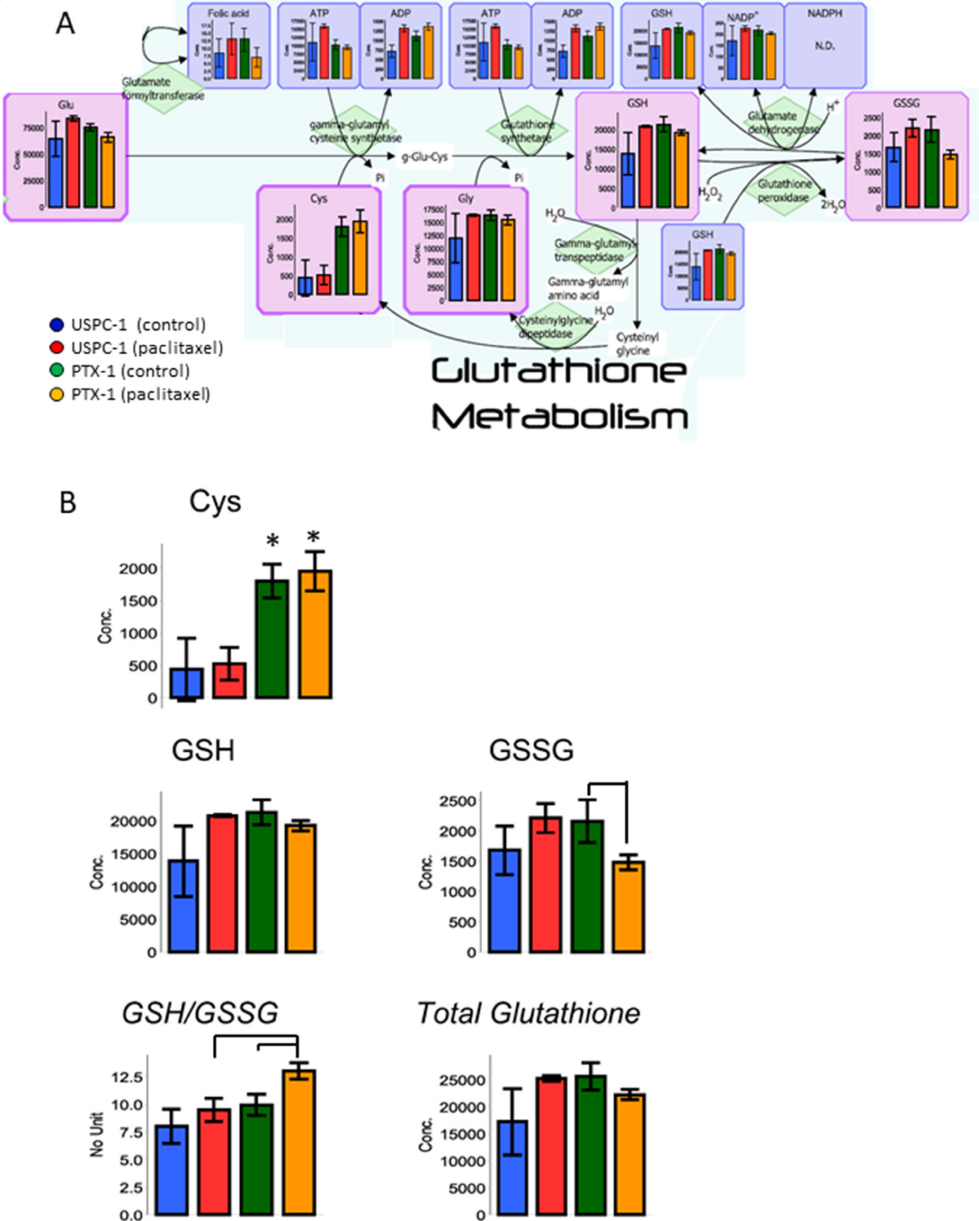
Glutathione (GSH) metabolism analysis after treatment with paclitaxel in uterine serous carcinoma cells (**A**) Each cell line was treated with 15 nM paclitaxel or control vehicle for 24 h. Blue bars represent USPC-1 cells (control), red bars represent USPC-1 cells treated with paclitaxel, green bars represent PTX-1 cells (control) and yellow bars represent PTX-1 cells treated with paclitaxel. Values in the graphs represent the means ± SD of three independent experiments. (**B**) Concentrations of cysteine, GSH, GSSG and total glutathione in USC cells after treatment with paclitaxel. GSH/GSSG (glutathione redox ratio) = [GSH]/[GSSG]. Total glutathione = [GSH] + 2 × [GSSG], ^*^P < 0.05. N.D.: not detected.

GSH is a tripeptide consisting of glutamic acid, cysteine and glycine. Cysteine and GSH concentrations in PTX-1 cells were higher than in USPC-1 cells (Table 1, Figure 5). GSH concentration in the USPC-1 cells increased after paclitaxel treatment but was unchanged in PTX-1 cells (Table 1, Figure5). This indicates that GSH may be related to paclitaxel resistance. In addition, the glutathione redox ratio (GSH/GSSG) in USPC-1 cells was unchanged, but was significantly elevated after paclitaxel treatment in PTX-1 cells (Table 1, Figure 5).

**Table 1:** Concentration of metabolites about glutathione metabolic pathways in USC cells

Compound name	Concentration (pmol/10^6^ cells)															
	USPC-1(Control)		USPC-1(Paclitaxel)		PTX-1(Control)		PTX-1(Paclitaxel)		USPC-1(Paclitaxel) vs USPC-1(Control)		PTX-1(Paclitaxel) vs PTX-1(Control)		PTX-1(Control) vs USPC-1(Control)		PTX-1(Paclitaxel) vs USPC-1(Paclitaxel)	
	Mean	S.D.	Mean	S.D.	Mean	S.D.	Mean	S.D.	Ratio	*P* value	Ratio	*P* value	Ratio	*P* value	Ratio	*P* value
Folic acids	8.5	4.5	13	5.2	13	3.8	7.1	3.3	1.5	0.313	0.5	0.113	1.5	0.261	0.5	0.178
ADP	716	159	1,319	98	1,118	125	1,364	89	1.8	0.009^*^	1.2	0.056	1.6	0.029^*^	1.0	0.588
ATP	11,072	5,718	15,846	651	10,294	1,417	9,452	631	1.4	0.284	0.9	0.422	0.9	0.838	0.6	2.6E-04^*^
Gly	11,954	4,697	16,374	245	16,379	1,012	15,489	914	1.4	0.244	0.9	0.322	1.4	0.241	0.9	0.231
**Cys**	436	482	519	251	1,796	262	1,950	304	1.2	0.851	1.1	0.545	4.1	0.112	3.8	0.004^*^
Glu	64,248	16,387	83,494	2,271	75,376	3,081	65,793	4,357	1.3	0.177	0.9	0.041^*^	1.2	0.360	0.8	0.008^*^
Glutathione (GSSG)	1,679	402	2,208	239	2,158	352	1,480	123	1.3	0.138	0.7	0.066	1.3	0.197	0.7	0.019^*^
**Glutathione (GSH)**	13,828	5,374	20,770	138	21,282	1,894	19,231	760	1.5	0.155	0.9	0.193	1.5	0.126	0.9	0.068
NADPH/NADP+	N.A.	N.A.	N.A.	N.A.	N.A.	N.A.	N.A.	N.A.	N.A.	N.A.	N.A.	N.A.	N.A.	N.A.	N.A.	N.A.

S.D.; standard deviation, ADP; adenosine diphosphate, ATP; adenosine triphosphate, NADPH; reduced nicotinamide adenine dinucleotide phosphate, NADP; nicotinamide adenine dinucleotide phosphate, ^*^P < 0.05, N.A.; not available

**Figure 6:**
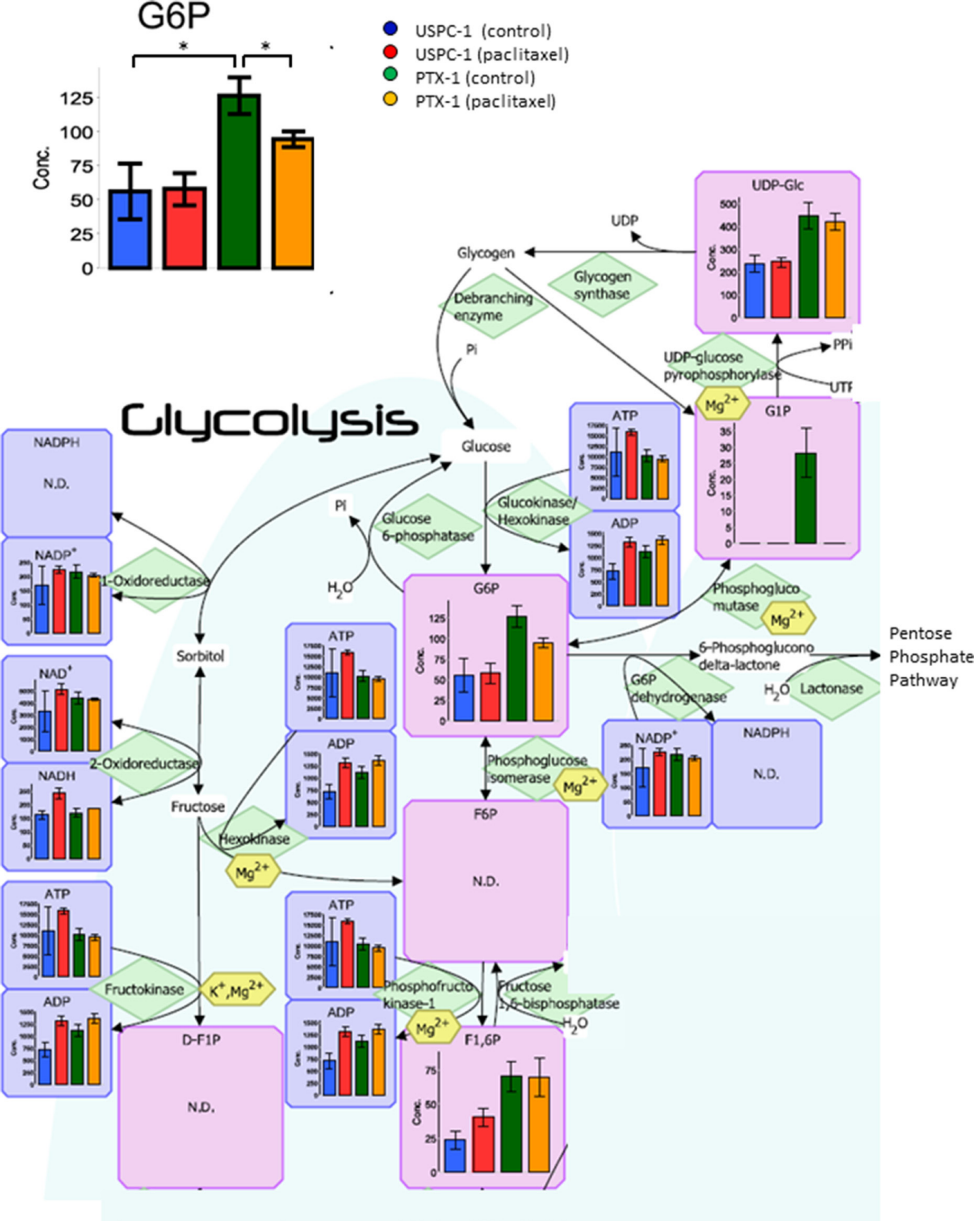
Analysis of the glycolytic pathway after treatment with paclitaxel in uterine serous carcinoma cells Each cell line was treated with 15 nM paclitaxel or control vehicle for 24 h. Blue bars represent USPC-1 cells (control), red bars represent USPC-1 cells treated with paclitaxel, green bars represent PTX-1 cells (control) and yellow bars represent PTX-1 cells treated with paclitaxel. Values in the graphs represent the means ± SD of three independent experiments. ^*^P < 0.05. N.D.: not detected.

**Figure 7:**
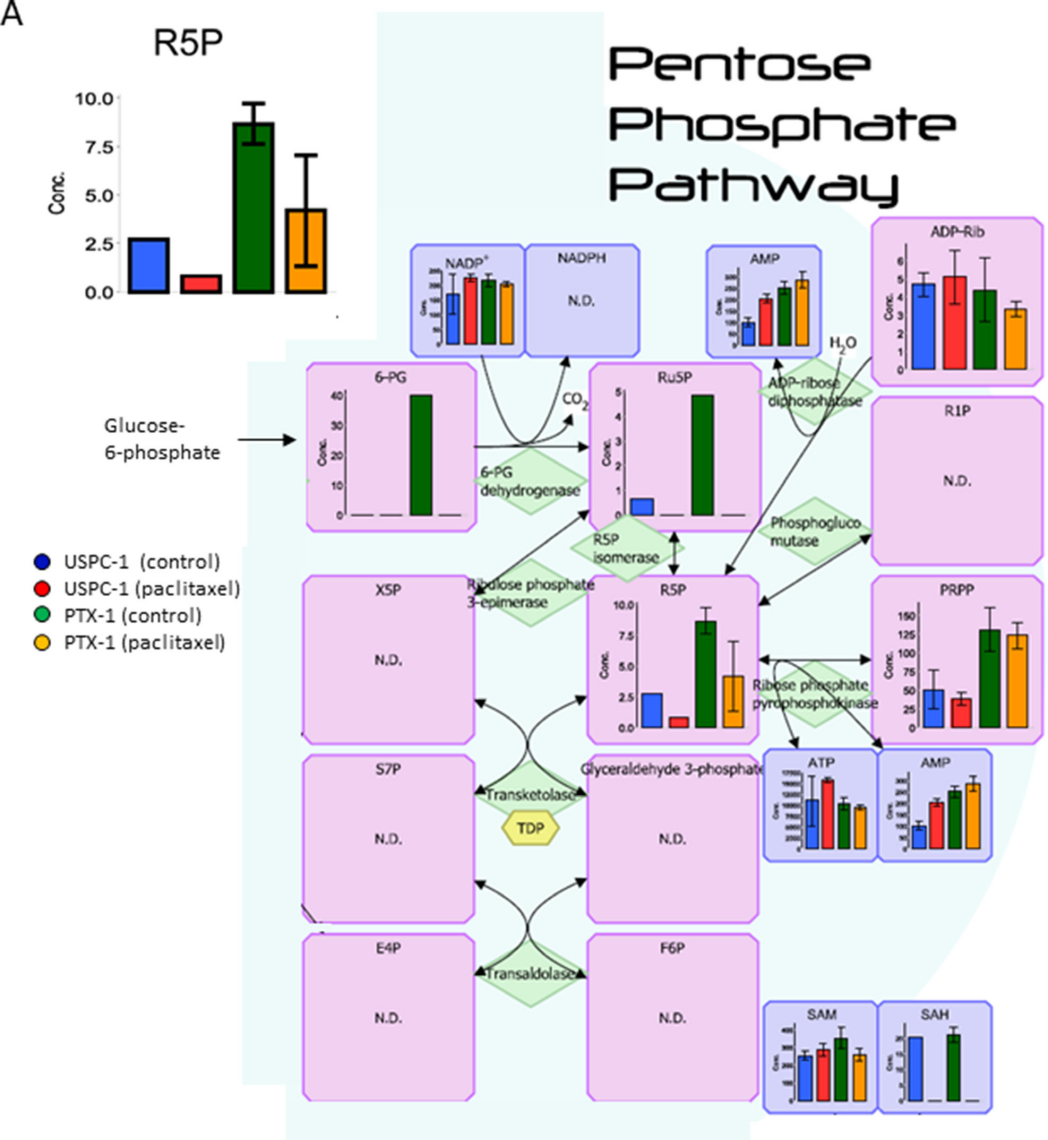
Analysis of the pentose phosphate pathway after treatment with paclitaxel in uterine serous carcinoma cells Each cell line was treated with 15 nM paclitaxel or control vehicle for 24 h. Blue bars represent USPC-1 cells (control), red bars represent USPC-1 cells treated with paclitaxel, green bars represent PTX-1 cells (control) and yellow bars represent PTX-1 cells treated with paclitaxel. Values in the graphs represent the means ± SD of three independent experiments. ^*^P < 0.05. N.D.: not detected.

**Figure 8:**
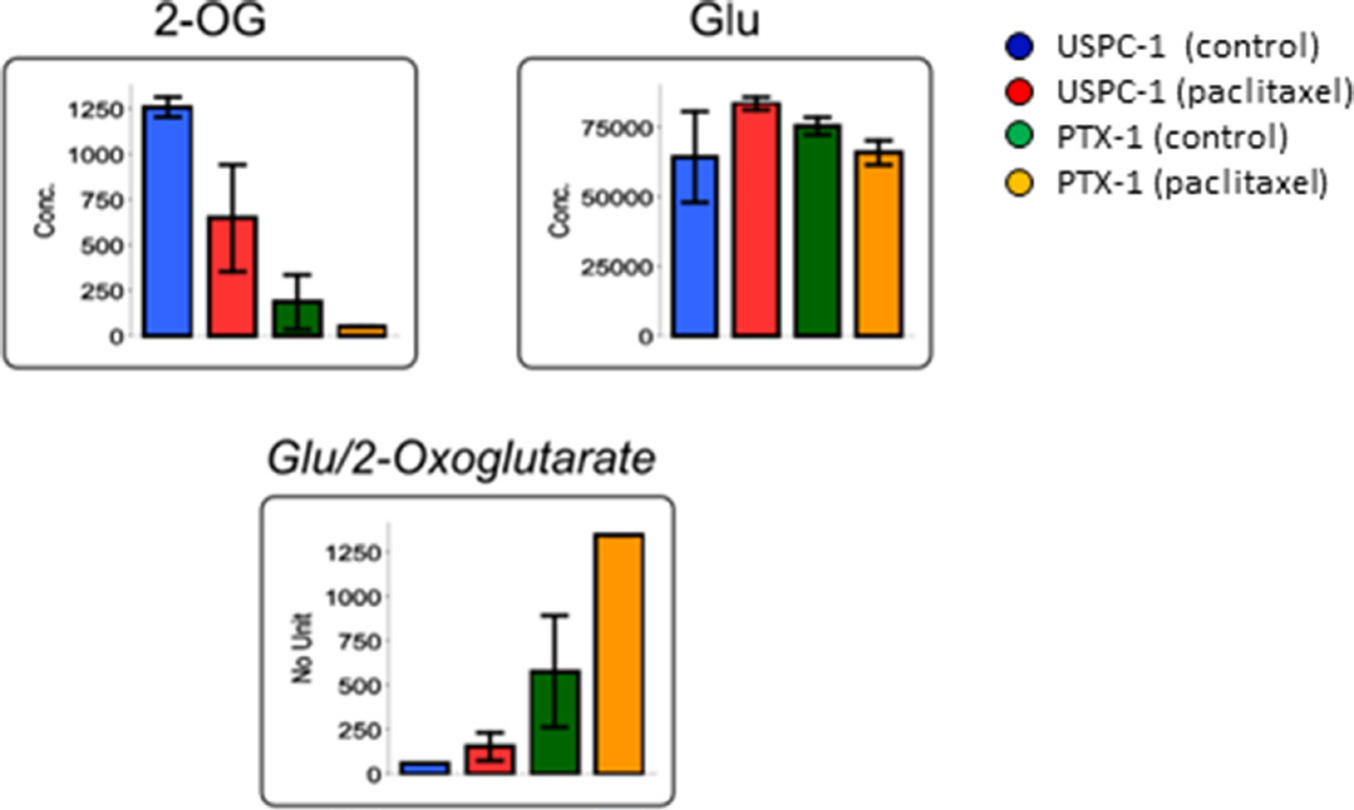
Concentration of 2-oxoglutarate (2-OG) and glutamic acid (Glu) after treatment with paclitaxel in uterine serous carcinoma cells Each cell line was treated with 15 nM paclitaxel or control vehicle for 24 h. Blue bars represent USPC-1 cells (control), red bars represent USPC-1 cells treated with paclitaxel, green bars represent PTX-1 cells (control) and yellow bars represent PTX-1 cells treated with paclitaxel. Values in the graphs represent the means ± SD of three independent experiments. ^*^P < 0.05.

**Figure 9:**
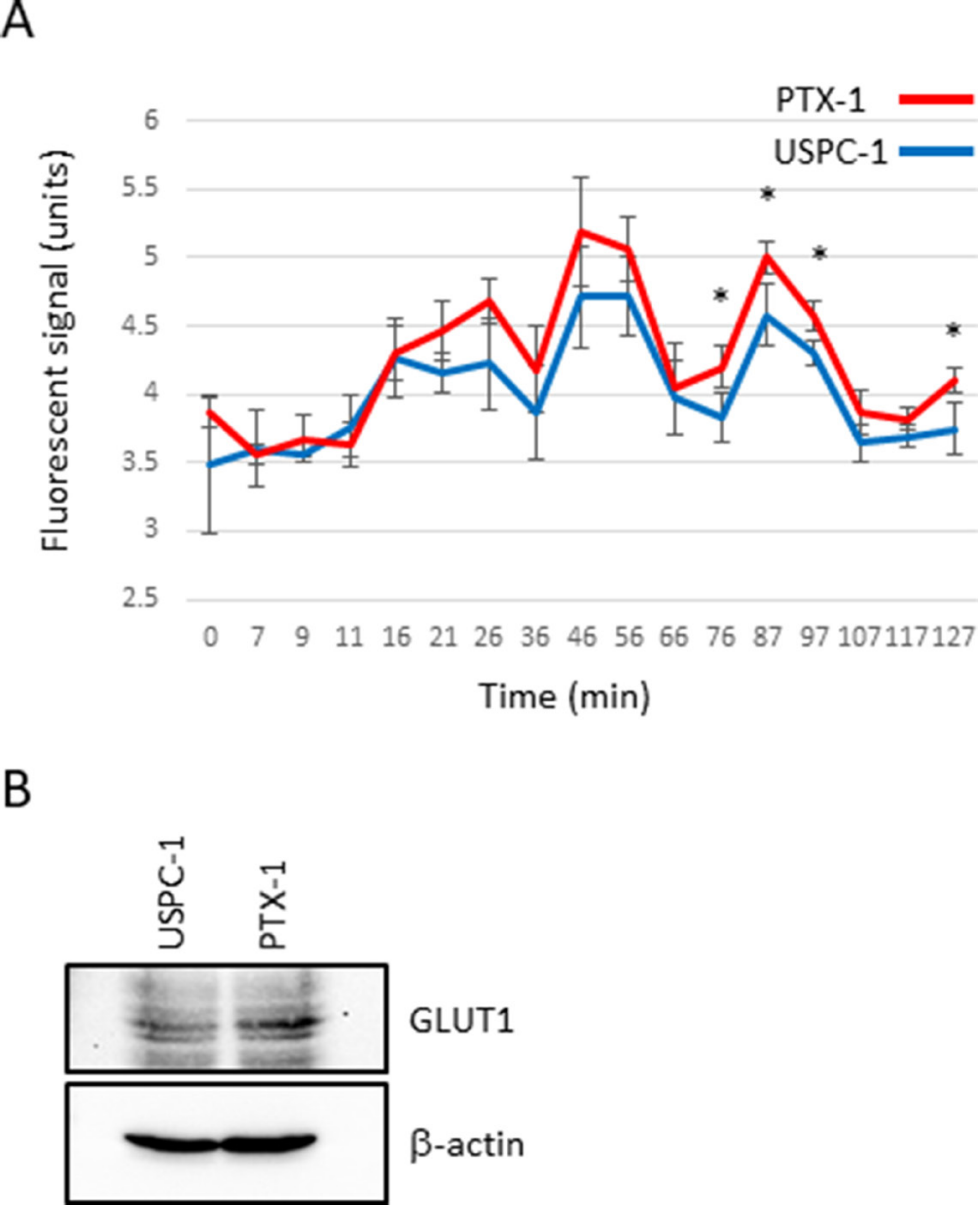
Glucose consumption and glucose transporter expression in uterine serous carcinoma cells (**A**) Oxygen consumption assay. USPC-1 and PTX-1 cells were seeded in a 96-well plate at density of 8.0 × 10^4^ cells. The plate was read using a fluorescence plate reader. The excitation and emission spectra were 380 nm and 650 nm, respectively. The values in the graphs represent means ± SD of three independent experiments. ^*^P < 0.05. (**B**) USPC-1 and PTX-1 cells were subjected to immunoblot analysis of GLUT1 and β-actin.

Next, we studied glucose metabolism in both cell lines. Glucose-6-phosphate (G6P) concentration in PTX-1 cells was higher than in USPC-1 cells (Table 2, Figure 6). G6P concentration in the USPC-1 cells was unchanged by paclitaxel treatment while it decreased in PTX-1 cells (Table 2, Figure 6). In the Pentose pathway, ribose-5-phosphate (R5P) and phosphoribosyl diphosphate (PRPP) concentrations in PTX-1 cells were higher than those in USPC-1 cells (Table 2, Figure 7). To examine glucose consumption in USC cells, we performed oxygen consumption tests in USPC-1 and PTX-1 cells. After about 60 min until treatment with the reagent, oxygen consumption in PTX-1 cells was higher than that in USPC-1 cells (Figure 9A). Additionally, GLUT1 expression in PTX-1 cells was higher than that in USPC-1 cells (Figure 9B). Finally, 2-oxoglutarate levels in USPC-1 cells were higher than in PTX-1 cells, and the ratio of glucose to 2-oxoglutarate in USPC-1 cells was lower than that in PTX-1 (Figure 8).

**Table 2:** Concentration of metabolites about glycolysis in USC cells

Compound name	Concentration (pmol/10^6^ cells)															
	USPC-1(Control)		USPC-1(Paclitaxel)		PTX-1(Control)		PTX-1(Paclitaxel)		USPC-1(Paclitaxel) vs USPC-1(Control)		PTX-1(Paclitaxel) vs PTX-1(Control)		PTX-1(Control) vs USPC-1(Control)		PTX-1(Paclitaxel) vs USPC-1(Paclitaxel)	
	Mean	S.D.	Mean	S.D.	Mean	S.D.	Mean	S.D.	Ratio	*P* value	Ratio	*P* value	Ratio	*P* value	Ratio	*P* value
NAD+	3,308	1,684	5,126	463	4,368	509	4,322	81	1.5	0.197	1.0	0.890	1.3	0.392	0.8	0.091
NADH	162	15	241	19	169	14	184	N.A.	1.5	0.006^*^	1.1	N.A.	1.0	0.598	0.8	N.A.
UDP-glucose	235	39	244	21	446	58	420	38	1.0	0.756	0.9	0.560	1.9	0.009^*^	1.7	0.005^*^
**G6P**	56	20	58	12	126	13	94	5.8	1.0	0.898	0.7	0.037^*^	2.3	0.001^*^	1.6	0.019^*^
**F1, 6P**	24	6.4	40	6.9	70	11	70	14	1.7	0.036^*^	1.0	0.980	3.0	0.006^*^	1.7	0.050
ADP	716	159	1,319	98	1,118	125	1,364	89	1.8	0.009^*^	1.2	0.056	1.6	0.029^*^	1.0	0.588
GTP	1,815	707	2,550	90	2,059	280	1,998	28	1.4	0.212	1.0	0.740	1.1	0.622	0.8	0.005^*^
ATP	11,072	5,718	15,846	651	10,294	1,417	9,452	631	1.4	0.284	0.9	0.422	0.9	0.838	0.6	2.6E-04^*^
NADPH/NADP+	N.A.	N.A.	N.A.	N.A.	N.A.	N.A.	N.A.	N.A.	N.A.	N.A.	N.A.	N.A.	N.A.	N.A.	N.A.	N.A.
NADH/NAD+	0.06	0.04	0.05	0.008^*^	0.04	0.005	0.04	N.A.	0.8	0.591	1.1	N.A.	0.6	0.426	0.9	N.A.
G6P/R5P	17	N.A.	89	N.A	15	2.9	29	15	5.1	N.A.	2.0	0.236	0.9	N.A.	0.3	N.A.

NAD; nicotinamide adenine dinucleotide, NADH; reduced nicotinamide adenine dinucleotide, G6P; glucose 6-phosphate, F1, 6P; fluctose 1,6-diphosphate, R5P; ribose 5-phosphate, NADPH; reduced nicotinamide adenine dinucleotide phosphate, NADP; nicotinamide adenine dinucleotide phosphate, S.D.; standard deviation, ^*^P < 0.05, N.A.; not available.

## DISCUSSION

USC is one of the most malignant types of gynecological malignancies. Most cases of USC relapse after the initial treatment, like epithelial ovarian serous carcinomas. Some studies suggested a beneficial role of adjuvant chemotherapy for USC even when diagnosed at an early stage [10–12]. We analyzed metabolites in USC cells to reveal the mechanisms of acquired resistance to paclitaxel.

Lipid metabolism is associated with cancer and particularly free fatty acids may be involved in the development of cancer [17]. Abnormal free fatty acid synthesis is among the prevalent features of various cancers, including ovarian cancer [18]. Malonyl-CoA in fatty acid synthesis mediates cell death in breast cancer cells [19]. In this analysis, malonyl-CoA levels in the paclitaxel sensitive USPC-1 cells are elevated after paclitaxel treatment. This result indicates that in USC cells, malonyl-CoA may be associated with paclitaxel-induced cell death.

It is known that creatinine kinase (CK) is related to sarcoma development. CK expression is decreased in malignant tissue compared to normal tissue [20]. Additionally, CK regulates the cell cycle in cancer cells [21]. Our study indicates that creatinine kinase is relevant for paclitaxel resistance, because the creatine concentration in the paclitaxel resistant cells is higher than in sensitive cells.

Methionine plays important roles in cancer cells by regulating cell death and survival [22], as well as metastasis in breast cancer [23]. Our data show that the methionine concentration in PTX-1 cells was higher than that in USPC-1 cells, and methionine levels were elevated after treatment by paclitaxel in USPC-1 cells. This indicates that methionine concentration is elevated after paclitaxel treatment and is associated with paclitaxel resistance. Cystathionine beta synthase is generally associated with drug resistance, including resistance to anti-tumor agents [24, 25]. In this analysis, cystathionine was elevated after paclitaxel treatment in USPC-1 cells, but not in PTX-1 cells. This suggests that cystathionine beta synthase in the USPC-1 cells was activated via pathways for expelling paclitaxel. On the other hand, cystathionine levels in PTX-1 cells did not change. We speculate that PTX-1 cells may expel paclitaxel via other mechanisms or that paclitaxel intake in PTX-1 cells may be blocked.

Glucose metabolism may also have important roles in the sensitivity of cancer cells to chemotherapeutics. It has been shown that cancer cells increase glucose uptake and glycolysis [26]. Therefore, the glucose metabolic pathway in cancer cells may be a target for cancer therapy [27]. Glucose is taken up into cells via the glucose transporter and is subsequently phosphorylated by hexokinase. We demonstrated that G6P and R5P concentrations in paclitaxel-resistant cells were higher than those in paclitaxel-sensitive cells; glucose consumption and GLUT1 expression in resistant cells were higher than those in sensitive cells. Cancer cells have been shown to shift their energy production from the oxidative phosphorylation pathway to glycolysis, known as the Warburg effect [28]. In contrast, it was reported that not all tumors shift to glycolysis for energy production; some diffuse large B-cell lymphomas and glioblastomas remain dependent on oxidative phosphorylation for energy production [29, 30]. Interestingly, oxidative phosphorylation and GLUT1 expression in ovarian cancer cells with acquired resistance were higher than those in sensitive cells. It was reported that cells with acquired resistance exhibited a high metabolically active phenotype with the ability to switch between oxidative phosphorylation and glycolysis compared with sensitive cells [31]. Our data show that glucose use shifts metabolism toward more oxidative phosphorylation and mitochondrial function in paclitaxel-resistant cells, and some studies reported the importance of mitochondrial function in enabling therapeutic resistance [32]. It is suggested that mitochondrial function may be a new therapeutic target in paclitaxel-resistant cells. In addition, 2-oxoglutarate (2-OG) is being produced from glutamic acid (Glu) via various amino acid metabolic pathways. Particularly, the ratio of Glu/2-OG is indicative for amino acid synthesis and degradation. The amino acid metabolism of paclitaxel resistant cells may be more activated than that of sensitive cells.

The concentration of glutathione in the paclitaxel-resistant cells was higher than that in paclitaxel-sensitive cells. GSH is a tripeptide consisting of glutamate, cysteine, and glycine. The GSH concentration in cancer cells is regulated by the cysteine transporter xCT [33]. xCT is stabilized by a variant form of CD44 which is associated with cancer stem cells [34]. GSH is one of the main antioxidants in cancer cells. Reactive oxygen species (ROS) are being produced by anticancer agents in cancer cells and can induce apoptosis [35]. As an antioxidant, GSH prevents the increase of ROS in cancer cells. We hypothesize that GSH prevents apoptosis induced by paclitaxel in paclitaxel resistant cells. In addition, the glutathione redox ratio (GSH/GSSG) indicates the oxidative stress status in cancer cells. With increasing oxidative stress in the cells, the ratio of GSH/GSSG decreases. In this study, the GSH/GSSG ratio in the paclitaxel sensitive cells was unchanged after paclitaxel treatment, but was elevated in the paclitaxel resistant cells. This result suggests that paclitaxel resistant cells have the ability to detoxify oxidative stress.

Many studies reported metabolomic analysis of various cancers, including ovarian cancer [16]. This is a very important avenue of research that may reveal specifically the reaction to anticancer agents and alterations of signaling pathways useful for cancer therapy. Metabolomic profiling analysis is a powerful tool to understand the biological pathways of cancer cells, and could detect organ- or cell-specific changes easily. However, the results might contain junk data, so it is necessary to verify the results from metabolomic profiling analysis. We focused on GSH and glycolysis from our data because these are associated with chemoresistance, include cancer stem cells [27, 34]. We are currently researching the GSH mechanism that confers chemoresistance in USC cells. We think metabolomic analysis is very important for seizing opportunities for cancer therapies, and this study is the beginning of comprehending the reaction of USC cells to paclitaxel, and identifying new molecular targets for therapy. This method is useful for explorative investigations to detect new therapeutic targets.

Our study indicates that an increased GSH and glucose metabolism may be relevant for acquiring resistance to paclitaxel in USC cells and thus these may be targets for anti-USC therapy.

## MATERIALS AND METHODS

### Cell culture

Human USC cell lines USPC-1 and PTX-1 were kindly provided by Dr. Santin, Department of Obstetrics and Gynecology, Division for Gynecologic Oncology at the Yale University School of Medicine (New Haven, CT, USA) [36]. PTX-1 was established from USPC-1 by maintaining the cells in medium with a low dose of paclitaxel for three months. The cells were maintained in RPMI1640 medium supplemented with 10% fetal bovine serum and GlutaMAX™ I (Thermo Fisher Scientific, Waltham, U.S.A.). The culture medium was supplemented with 100 U/ml penicillin and 100 μl streptomycin and changed every 3 days.

### Reagents

Paclitaxel was purchased from Sigma-Aldrich (St. Louis, MO, USA) and dissolved in dimethyl sulfoxide (DMSO) to prepare a 10 mM stock solution. D-mannitol and methanol were purchased from Wako (Osaka, Japan). Anti-glucose transporter 1 (GLUT1) antibody (sc-7903) was purchased from Santa Cruz Biotechnology, Inc. (Santa Cruz, CA, USA). Anti-β-actin antibody (A1978) was purchased from Sigma-Aldrich (St. Louis, MO, USA).

### Cell viability assay

Viable and dead cells were identified by their ability and inability to retain trypan blue [37, 38]. Cells were stained with 0.2% trypan blue, and the numbers of viable and dead cells were determined using a hemocytometer. Cell viability (%) was defined as 100 × (number of viable cells/number of total cells), whereas the percentage of dead cells was defined as 100 × (number of dead cells/number of total cells). To determine the IC_50_ values of paclitaxel for USPC-1 cells used in the present study, we treated the cells with varying concentrations of paclitaxel for 24 h and then determined their viability. The IC_50_ values were calculated using the following fomula [39]:

IC_50_ = 10^[log(A/B)×(50-C)]/[(D-C)+log(B)]^ where A and B are the corresponding concentrations of the tested drug directly above and below 50% inhibition, respectively, and C and D correspond to the percentage of inhibition directly below and above 50% inhibition, respectively.

### Metabolomic analysis

Metabolomic analysis was performed by Human Metabolome Technologies (HMT Inc., Tsuruoka, Japan) as described previously [40]. The cells were treated with paclitaxel (15 nM) for 24 h. Metabolites were then extracted from 3–4.5 × 10^6^ cells with methanol containing Internal Standard Solution (HMT Inc., Tsuruoka, Japan) and analyzed using a capillary electrophoresis CE-MS/MS system (HMT Inc., C-SCOPE). Culture medium was removed from a 10-cm culture dish and cells were washed twice in 5% mannitol solution (first with 10 mL and then with 2 mL) within 2 min. Next, cells were treated with 800 μL methanol for 30 s in order to inactivate enzymes, and 550 μL Milli-Q water containing internal standards (H33304-1002, HMT Inc.) for another 30 s. The extract was transferred into a microfuge tube and centrifuged at 2,300 × *g* and 4° C for 5 min. Then, the upper layer was centrifugally filtered through a Millipore 5-kDa cutoff filter at 9,100 × *g* and 4° C for 120 min to remove proteins. The filtrate was centrifugally concentrated and resuspended in 50 μL of Milli-Q water. Peaks detected by CE-TOFMS and CE-MS/MS were extracted using automatic integration software (MasterHands, Keio University, Tsuruoka, Japan and MassHunter Quantitative Analysis B.04.00, Agilent Technologies, Santa Clara, CA, USA, respectively) [41]. The peaks were annotated with putative metabolites from the HMT metabolite database based on their migration times (MTs) in CE and *m/z* values determined by TOFMS and MS/MS. The tolerance range for the peak annotation was configured at ± 0.5 min for MT and ± 10 ppm for *m/z*. In addition, concentrations of metabolites were calculated by normalizing the peak area of each metabolite with respect to the area of the internal standard and by using standard curves, which were obtained by three-point calibrations.

### Oxygen consumption assay

To examine glucose consumption in USC cancer cells, we used an oxygen consumption assay kit (MitoXpress Xtra^®^ Oxygen Consumption Assay; #26140-60-3) purchased from Lucxel Biosciences Ltd. (Lucxel Biosciences Ltd., Cork, Ireland). The cells were seeded in a 96-well plate at a density of 8.0 × 10^4^ cells in each well in 200 μL of culture medium. The cells were incubated for 3 h, and were adhered to the plate; culture medium was then removed and 90 μL of fresh, warmed culture medium and 10 μL of MitoXpress Xtra^®^ reagent were added. Each well was sealed using mineral oil. Immediately, the plate was read using a fluorescence plate reader (Thermo Scientific VarioSkan^®^ Flash, Thermo Fisher Scientific, Waltham, USA.). The excitation and emission spectra were 380 nm and 650 nm, respectively.

### Immunoblot analysis

Immunoblot analysis was performed as described previously [27]. Cells were washed with ice-cold phosphate-buffered saline (PBS) and lysed in radioimmunoprecipitation assay buffer [10 mM Tris-HCl (pH 7.4), 0.1% sodium dodecyl sulfate (SDS), 1% sodium deoxycholate, 150 mM NaCl, 1 mM ethylenediaminetetraacetic acid, 1.5 mM Na_3_VO_4_, 10 mM NaF, 10 mM sodium pyrophosphate, 10 mM sodium β-glycerophosphate and 1% protease inhibitor cocktail set III (Calbiochem)]. After centrifugation for 10 min at 14,000 × *g* at 4° C, the supernatants were recovered as the cell lysates; the protein concentrations of these cell lysates were determined using the bicinchoninic acid protein assay kit (Pierce Biotechnology, Inc., Rockford, IL, USA). Cell lysates containing equal amounts of protein were separated using SDS-polyacrylamide gel electrophoresis and transferred to a polyvinylidene difluoride membrane. The membrane was probed with a primary antibody and then with an appropriate horseradish peroxidase (HRP)-conjugated secondary antibody according to the protocol recommended by the manufacturer of each antibody. Immunoreactive bands were visualized using Amersham™ ECL™ Prime Western Blotting Detection Reagent (GE Healthcare UK Ltd., Buckinghamshire, England).

### Flowcytometric analysis

Dissociated cells were washed with ice-cold PBS, and fixed with ethanol at a final concentration of 70% ethanol. After storage overnight at –25° C, the cells were washed with PBS twice at room temperature. The cells were treated with ribonuclease (30 mg/mL) for 30 min, and then treated with propidium iodide. All flow cytometric experiments were run using the FACSCanto™ II Flow Cytometer (BD Biosciences, Franklin Lakes, NJ, USA) and the data were analyzed using FlowJo software, version 7.6.5 (Treestar Inc., Ashland, OR, USA).

### Statistical analysis

Results are expressed as the means ± standard deviations (SD), and differences were compared using the two-tailed Student's *t*-test. *P* < 0.05 was considered to indicate a statistically significant difference and is denoted using asterisks in the figures.

## SUPPLEMENTARY MATERIALS FIGURES AND TABLES


