# Correction: Metabolomic analysis of uterine serous carcinoma with acquired resistance to paclitaxel

**DOI:** 10.18632/oncotarget.27474

**Published:** 2021-11-09

**Authors:** Manabu Seino, Tsuyoshi Ohta, Akiko Sugiyama, Hirotsugu Sakaki, Takeshi Sudo, Seiji Tsutsumi, Shogo Shigeta, Hideki Tokunaga, Masafumi Toyoshima, Nobuo Yaegashi, Satoru Nagase

**Affiliations:** ^1^ Department of Obstetrics and Gynecology, Yamagata University School of Medicine, Iidanishi, Yamagata 990-9585, Japan; ^2^ Department of Obstetrics and Gynecology, Tohoku University Graduate School of Medicine, 1-1 Seiryo-machi, Aoba-Ku, Sendai, Miyagi, 980-8574, Japan

**This article has been corrected:** Due to errors during figure preparation, Figure 5 was marked and labeled incorrectly. Specifically, the value of cysteine has been corrected in the first column of the box labeled ‘Cys’ in Figure 5B (USPC-1 Control). Lines and asterisks denoting comparisons between groups were also added to this box. X-axis lines were added to all boxes in 5B, along with concentration units on the Y axis. The color key used in 5A has also been added to 5B. The proper Figure 5 is shown below. In addition, the information for affiliation 2 is listed incorrectly; the proper affiliation is shown below. The authors declare that these corrections do not change the results or conclusions of this paper.

^2^Department of Obstetrics and Gynecology, Tohoku University Graduate School of Medicine, 1-1 Seiryo-machi, Aoba-Ku, Sendai, Miyagi, 980-8574, Japan

Original article: Oncotarget. 2018; 9:31985–31998. 31985-31998. https://doi.org/10.18632/oncotarget.25868

**Figure 5:**
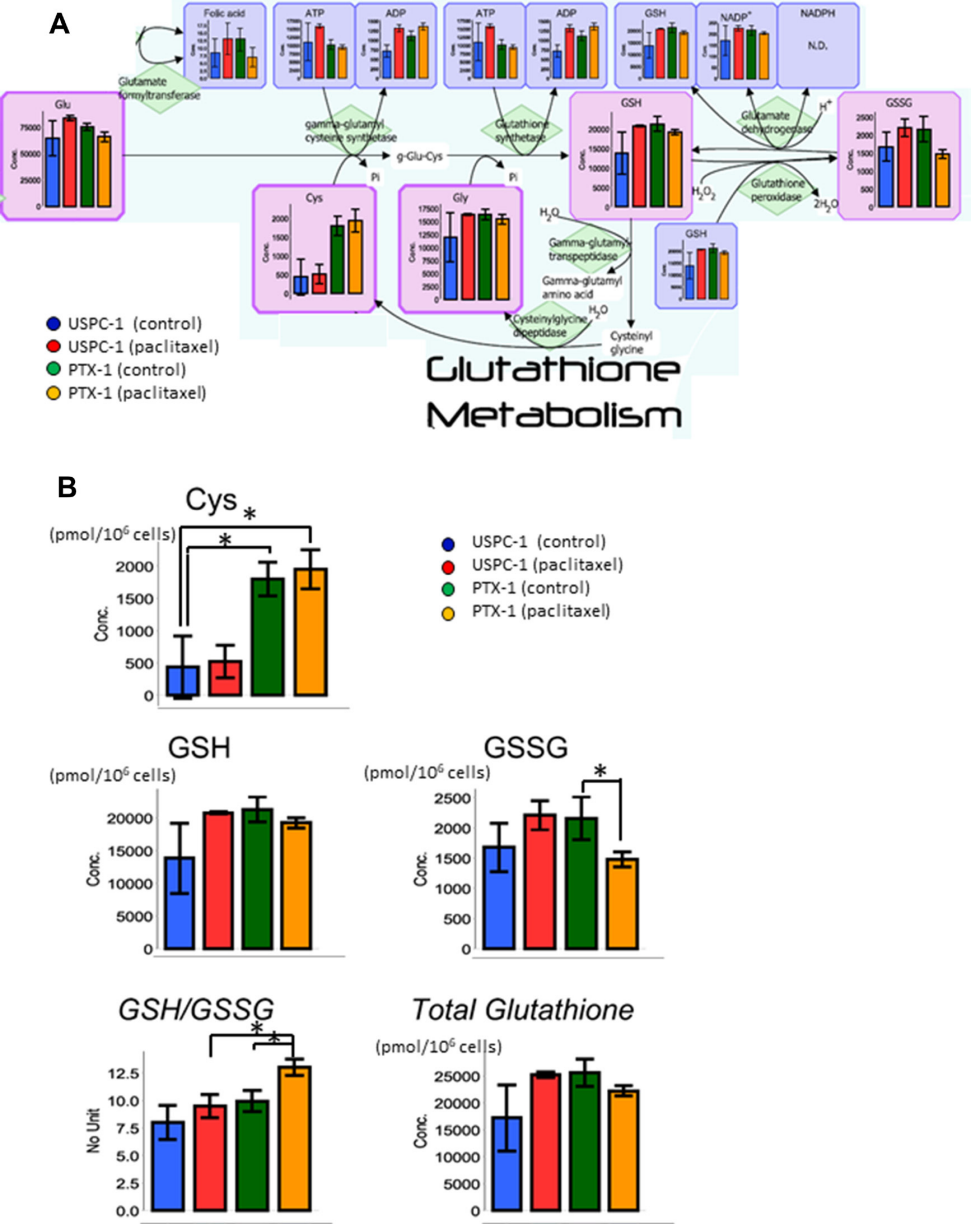
Glutathione (GSH) metabolism analysis after treatment with paclitaxel in uterine serous carcinoma cells. (**A**) Each cell line was treated with 15 nM paclitaxel or control vehicle for 24 h. Blue bars represent USPC-1 cells (control), red bars represent USPC-1 cells treated with paclitaxel, green bars represent PTX-1 cells (control) and yellow bars represent PTX-1 cells treated with paclitaxel. Values in the graphs represent the means ± SD of three independent experiments. (**B**) Concentrations of cysteine, GSH, GSSG and total glutathione in USC cells after treatment with paclitaxel. GSH/GSSG (glutathione redox ratio) = [GSH]/[GSSG]. Total glutathione = [GSH] + 2 × [GSSG], ^*^ *P* < 0.05. N.D.: not detected.

