# Supplementary material for: Metabolomic analysis of uterine serous carcinoma with acquired resistance to paclitaxel
Source: Oncotarget. 2018 Aug 10;9(62):31985–98. doi: 10.18632/oncotarget.25868 (PMC6112827; doi:10.18632/oncotarget.25868)
Supplement: Supplementary file 1 [file oncotarget-09-31985-s001.pdf]

Metabolomic analysis of uterine serous carcinoma with acquired resistance to paclitaxel

SUPPLEMENTARY MATERIALS

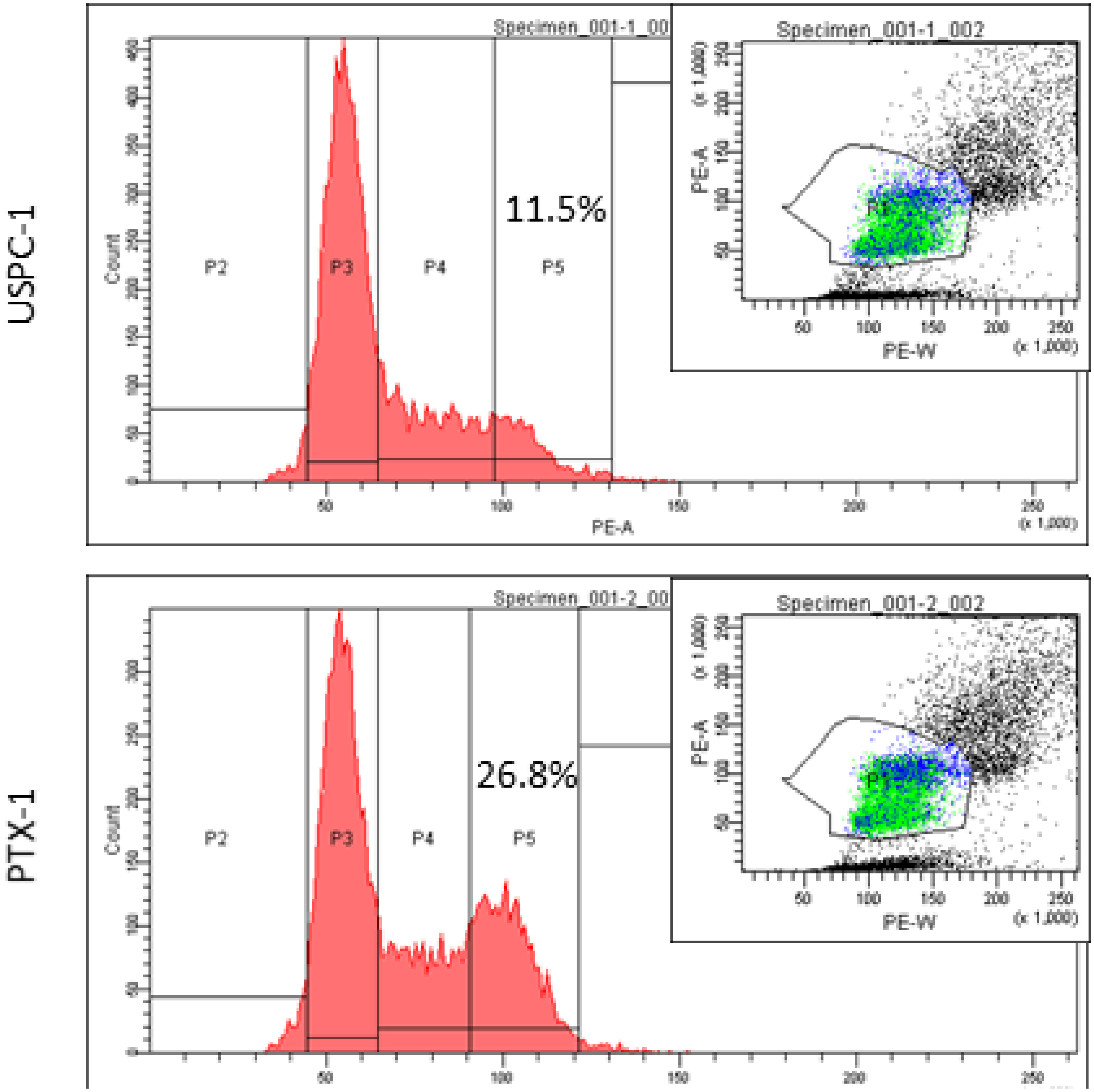

Supplementary Figure 1: Cell cycle analysis in uterine serous carcinoma cells. USPC-1 and PTX-1 cells were subjected to flowcytometric analysis. Representative histograms with the proportion of the cell population in the G2/M phase in USPC-1 and PTX-1 cells are shown.
